# Differential expression of platelet CD147 in moderate altitude conditions: implications for coronary plaque stability assessment

**DOI:** 10.3389/fcvm.2025.1621491

**Published:** 2025-10-27

**Authors:** Yu Chen, Si Lu, Chun-Ping Bao, Yong Ren, Juan Lv, Si-Jie Wang, Li-Sha Wang, Rui-Xi Zi, Xin Zhang, Li-Xia Yang, Yan Wang, Yan-Kun Shi

**Affiliations:** ^1^ Department of Cardiology, 920th Hospital of Joint Logistics Support Force, Kunming, China; ^2^ Department of Cardiology, Fuwai Yunnan Hospital, Chinese Academy of Medical Sciences, Affiliated Cardiovascular Hospital of Kunming Medical University, Kunming, China; ^3^ Department of Clinical Medical College, Dali University, Dali, China; ^4^ Department of Pulmonary and Critical Care Medicine, 920th Hospital of Joint Logistics Support Force, Kunming, China; ^5^ Department of Cardiology, First Affiliated Hospital of Kunming Medical University, Kunming, China

**Keywords:** CD147, basigin, acute coronary syndrome, coronary plaque stability, platelet function, bioinformatics analysis, clinical investigation

## Abstract

**Background:**

As a major contributor to global death rates, coronary heart disease (CHD) remains critical. This investigation examines CD147's utility for assessing plaque characteristics among CHD patients, with a focus on moderate altitude populations.

**Methods:**

Initially, high-throughput sequencing was performed on platelet samples obtained from three individuals with stable angina (SA) and three with unstable angina (UA), serving as the discovery cohort for subsequent bioinformatics analysis. Based on the insights gained from this initial phase, the investigation was extended to a larger cohort comprising 90 SA patients and 90 individuals diagnosed with acute coronary syndrome (ACS). Platelets isolated from these patients underwent flow cytometry analysis for CD147 expression. Furthermore, a logistic regression analysis incorporating traditional CHD risk factors was conducted to determine the odds ratio (OR) for CD147 expression in differentiating between stable and unstable plaques.

**Results:**

High-throughput sequencing revealed distinct CD147 levels between SA and UA groups (log₂FC = 2.3). The logistic regression analysis demonstrated that heightened platelet CD147 correlated with plaque instability (OR = 9.21, 95% CI: 2.33–36.42, *P* = 0.002), persisting beyond conventional risk adjustment.

**Conclusion:**

This study identifies platelet CD147 as a promising predictor for differentiating plaque stability in CHD under moderate altitude conditions. Our findings suggest that CD147 not only contributes to platelet activation and thrombus formation but may also directly influence plaque stability. The impact of chronic hypoxia and other environmental stressors in moderate altitude regions on CD147 expression provides new insights for risk stratification and targeted therapeutic strategies in altitude-specific populations.

## Introduction

Coronary artery atherosclerosis, a principal cause of global mortality, warrants particular attention to acute myocardial infarction (AMI) owing to its high incidence. AMI primarily results from unstable plaque rupture and subsequent thrombosis formation (1). These unstable plaques, with their thin fibrous caps and lipid-rich cores, show higher rupture risk than stable plaques, which contain more smooth muscle cells and collagen fibers (2, 3).While current techniques including Intravenous Ultrasound (IVUS), optical coherence tomography (OCT) (4), and biomarkers such as C-reactive protein (CRP) and interleukin-6 (IL-6) (5, 6) aid in plaque assessment, limitations in invasiveness and molecular specificity necessitate new evaluation approaches. This underscores the need for novel approaches to evaluate plaque stability more accurately.

CD147, as a highly glycosylated transmembrane protein, plays multiple roles in cardiovascular pathophysiology. Within platelets, CD147 is primarily stored in alpha granules (7) and demonstrates dynamic expression patterns during platelet activation (8). Our previous study revealed a crucial association between elevated platelet CD147 expression and plaque instability, independent of conventional risk factors, while monocyte CD147 levels remained unchanged (9). Despite these promising findings regarding CD147's role in cardiovascular conditions, two major limitations existed: the bioinformatics analysis utilized whole blood samples instead of platelet-specific specimens, and the study population was relatively small (31 stable angina patients and 30 acute coronary syndrome patients).

Beyond these molecular insights, environmental and geographic factors may significantly influence platelet heterogeneity and plaque stability in coronary artery disease (10). To address our previous study limitations and explore novel environmental influences, we focused on populations in moderate altitude regions (1,000–2,700 meters above sea level). These regions present distinct physiological challenges, including chronic hypoxia, reduced atmospheric pressure, and increased ultraviolet radiation exposure. Such environmental factors potentially modulate vascular physiology, platelet function, and inflammatory responses, ultimately affecting plaque stability (11). Of particular interest, hypoxic conditions have been demonstrated to enhance oxidative stress and alter platelet activation pathways—key mechanisms in cardiovascular pathology. However, how these unique environmental stressors modulate the relationship between platelet CD147 expression and plaque stability is not well-documented (12). Therefore, the primary aim of our study is to be the first to characterize the association between platelet CD147 expression and plaque stability specifically within this understudied moderate altitude population, thereby providing foundational insights in a unique physiological context.

**Figure 1:**
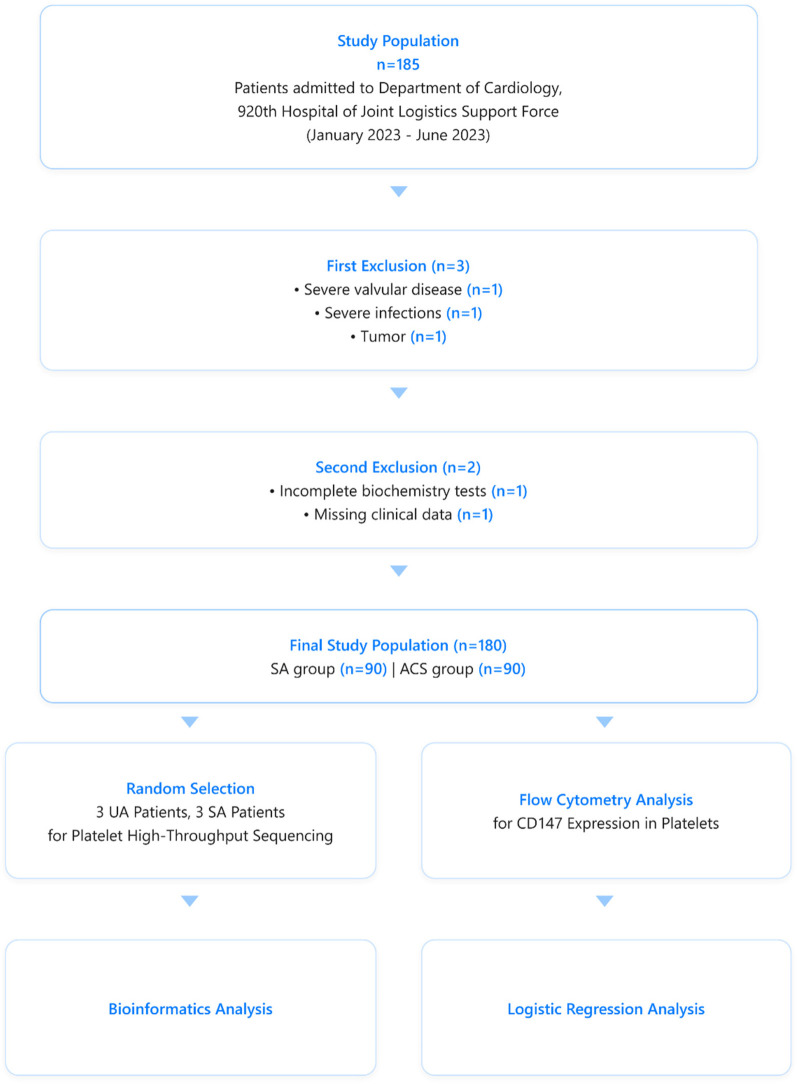
Patients selection and analytical flowchart for the comparative analysis of CD147 expression on platelets in stable angina (SA) and acute coronary syndrome (ACS) patients.

This study first employed high-throughput sequencing and bioinformatics to analyze platelet transcriptome differences between SA and UA patients. Based on these initial findings, we then utilized flow cytometry to investigate platelet CD147 expression in a larger cohort of SA and ACS patients (Figure 1). By integrating these analyses, we investigated platelet CD147's biological functions in plaque stability. This comprehensive approach provided expression data at the cellular level and revealed the variability of CD147 on platelets in different coronary heart disease states. Including moderate altitude conditions as a variable in this study provides a novel opportunity to investigate CD147's role in coronary artery disease under diverse environmental contexts, potentially offering new insights into its diagnostic and therapeutic value.

## Methods

### Study design and setting

A total of 180 patients (aged 20–75 years) who underwent coronary angiography were included in this cross-sectional study conducted at the Cardiology Department of the 920th Hospital during the period from January to June 2023.

### Participants

Inclusion criteria: (1) CHD diagnosis according to ACC/AHA standards (13); (2) Ability to undergo coronary angiography; (3) Initial CHD diagnosis; (4) Complete clinical data availability; (5) Age 20–75 years; (6) Local residency at 1,800–2,200 m altitude for extended period.

Exclusion criteria: (1) Major conditions such as heart failure, congenital or valvular heart disease, infections, hepatic/renal impairment, or malignancies; (2) Ineligibility for antiplatelet or anticoagulant use due to active ulcers, thrombocytopenia, coagulopathies, or bleeding tendencies; (3) Evidence of coronary slow flow during angiography.

The study enrolled 90 SA and 90 ACS patients. All participants provided informed consent. The study protocol followed the 2013 Declaration of Helsinki and received approval from our hospital's ethics committee.

### Variants

#### Baseline data

Demographic and clinical characteristics collected included age, gender, BMI, smoking status, and medical history (hypertension and diabetes).

#### Laboratory measurements

Blood specimens (5 ml) were obtained upon admission (within 4 h for stable angina; immediately for acute coronary syndrome). The analysis included hematological parameters (monocyte ratio and count, platelet indices), cardiac biomarkers (BNP, cTnI, CK, CK-MB), metabolic indicators (glucose, uric acid, Cr, homocysteine), inflammatory markers (CRP), lipid profile (triglycerides, cholesterols), and hepatic function tests (bilirubin fractions, *γ*-GT, cystatin C).

#### Coronary angiography

Trans-radial catheterization employed Seldinger's method with a 6F sheath, followed by multi-angle imaging of bilateral coronary vessels. Senior interventionalists (>10 years’ experience) evaluated the images through both qualitative assessment and quantitative computer analysis. Significant stenosis was defined as luminal diameter reduction ≥50%, with subsequent IVUS (Intravascular Ultrasound) examination targeting the most critical lesion.

#### IVUS examination for plaque characterization

Post-angiography, Virtual Histology-Intravascular Ultrasound (VH-IVUS) imaging was implemented under cardiac monitoring for detailed plaque characterization. To ensure consistency and eliminate inter-observer variability, all VH-IVUS images were analyzed by a single, senior interventional cardiologist with over 10 years of experience. Furthermore, to minimize potential intra-observer variability, the classification of plaque vulnerability was not based on subjective assessment but on strict adherence to the predefined, quantitative criteria: (1) three consecutive frames showing plaque burden exceeding 40%, with a necrotic core >10% in contact with the lumen; or (2) a necrotic core occupying >10% of the plaque area with a luminal contact angle greater than 30° (14, 15). This screening protocol enabled differentiation between stable (SA) and vulnerable (ACS) plaques.

#### Platelet RNA high-throughput sequencing

Six subjects (3 SA, 3 UA) were randomly selected for transcriptome profiling. The entire process of platelet isolation, RNA extraction, library construction, and sequencing was performed by Xishan District Juruo Experimental Supplies Business Department (Kunming, China) as a comprehensive service. According to their standard operating procedure, whole blood (10 ml) collected in EDTA tubes was processed immediately. First, blood was centrifuged at 120 g for 20 min (RT) to obtain platelet-rich plasma (PRP). To prevent ex vivo activation, Prostaglandin I₂ (PGI₂, final concentration 1 µM) (Beyotime, China) was added to the PRP. The PRP was then passed through a leukocyte-depletion filter (Pall, USA). The filtered PRP was subsequently centrifuged at 360 g for 20 min (RT) to pellet the platelets. The final platelet pellet was immediately resuspended in RNALater™ stabilizer (Beyotime, China) and stored at −80°C. The service provider confirmed the purity of isolated platelets by flow cytometry (<0.1% CD45-positive cells). Platelet RNA was isolated via RNA/DNA Co-extraction Kit (Beyotime, China) and quality-assessed using Bioanalyzer RNA picochip (Agilent, USA). Qualified RNA samples were used for library construction (TruSeq RNA kit, Illumina) and sequenced on a NextSeq 500 platform (Illumina, USA).

### Bioinformatics analysis

#### Data processing and quality control

Raw sequencing reads were first assessed for quality using FastQC (v0.11.9). Subsequently, Trimmomatic (v0.39) was used to remove adapter sequences, trim low-quality bases, and filter out short reads. The resulting high-quality reads were aligned to the human reference genome (GRCh38) using STAR (v2.7.9a), and gene-level counts were generated with featureCounts (v2.0.1). All samples passed quality control and were included in the subsequent differential expression analysis.

#### Differential gene expression

DESeq2 (v1.42.0, R 4.2.0) was employed to detect differentially expressed genes using thresholds of *P* < 0.05 and |log2FC| ≥ 2.26 for downstream analyses.

#### Functional enrichment analysis

To explore the biological functions and pathways associated with differentially expressed genes between different groups, we performed functional enrichment analysis using the clusterProfiler package (v 4.12.6) (16). Gene Ontology (GO) enrichment analysis (17) was conducted across three categories: biological process (BP), molecular function (MF), and cellular component (CC). KEGG pathway analysis (17) was also conducted to identify significantly enriched pathways. In both GO and KEGG analyses, terms with an adjusted *p*-value below 0.05 were deemed statistically significant.

Gene Set Enrichment Analysis (GSEA) was conducted using the clusterProfiler package to identify significantly enriched gene sets across groups. We utilized the MSigDB (Molecular Signatures Database) collections, including hallmark gene sets and KEGG pathway sets. Gene sets were defined as significantly enriched if they had a normalized enrichment score (NES) greater than 1 or less than −1, along with a false discovery rate (FDR) below 0.25.

Protein-Protein Interaction Network Construction. STRING database v12.0 (18) was utilized for protein interaction network analysis.

### Flow cytometry analysis

Venous blood (5 ml) was collected in 3.2% sodium citrate tubes (1:9 ratio) within 4 h of admission, or immediately for ACS cases. Platelet-rich plasma (PRP) was prepared by centrifuging whole blood at 300 g for 10 min at room temperature (RT) without brake. The PRP was carefully collected. To prevent procedural activation, PGI₂ was added to the PRP. The platelets were then washed through three sequential cycles. Each cycle consisted of centrifugation at 360 g for 20 min to pellet the platelets, removal of the supernatant, and gentle resuspension in a modified Tyrode's buffer containing PGI₂. This extensive washing process was designed to effectively remove residual plasma and potential contaminants. After the final wash, an aliquot of the platelet suspension was stained with an anti-CD45 antibody (eBioscience, USA) to confirm the absence of significant leukocyte contamination via flow cytometry. Finally, the stained samples were fixed with 1% paraformaldehyde before analysis on a flow cytometer.

Platelets were dual-labeled with fluorescein isothiocyanate (FITC)-CD147 and phycoerythrin (PE)-CD61 antibodies (eBioscience, USA). The specificity of the antibody binding was verified using appropriate isotype controls. Additionally, fluorescence-minus-one (FMO) controls were employed to establish accurate gating strategies and minimize background noise.

Platelet surface CD147 was quantified by Relative Fluorescence Intensity (RFI), compared with standard control samples, using EPICS XL flow cytometer (Coulter, USA) with daily calibration using standardized beads.

### Measurement standards for variables

#### Baseline data

Baseline clinical data were collected by qualified physicians according to standardized diagnostic criteria. Type 2 diabetes was diagnosed according to the Chinese Type 2 Diabetes Prevention and Treatment Guidelines (19), which require typical symptoms alongside one of the following: random venous plasma glucose ≥11.1 mmol/L; fasting venous plasma glucose ≥7.0 mmol/L; or 2-hour Oral Glucose Tolerance Test (OGTT) plasma glucose ≥11.1 mmol/L. Hypertension was diagnosed according to the National Guideline for Hypertension Management in China (2019) (20), defined as systolic blood pressure ≥140mmHg and/or diastolic pressure ≥90mmHg on repeated measurements on different days in the absence of antihypertensive medications. A history of smoking was defined as continuous or accumulated tobacco use for at least 6 months, or a lifetime intake of 100 or more cigarettes. Body mass index (BMI) was determined by dividing weight in kilograms by the square of height in meters.

#### Laboratory biochemical indicators

Biochemical measurements were performed in the clinical laboratory of the 920 Hospital.

#### Bias

To minimize potential bias, several measures were implemented: (1) Rigorous quality checks and instrument calibrations were performed prior to CD147-related experiments; (2) IVUS was employed for objective plaque stability assessment. (3) Considering the potential influence of antiplatelet medications (such as aspirin, clopidogrel) and lipid-lowering drugs (such as fluvastatin) on CD147 expression, detailed records of participants’ medication use were maintained. In the analysis of baseline data, we compared the differences in the use of these drugs between SA and ACS patients to control for confounding bias.

#### Sample size

Following the “events per variable” (EPV) rule of 10:1, logistic regression models require at least 10 events per variable to ensure statistical validity (21). With 90 positive events, we could include up to 9 variables in the model. According to empirical guidelines for sample size determination in regression analyses, the recommended subject-to-variable ratio ranges from 5:1 to 20:1.Five times is often considered acceptable, while 20 times aligns with the most stringent empirical rule (22). With 8 variables, the required sample size ranges from 40 to 160. Our total sample size of 180 meets this criterion, ensuring sufficient power for the analysis.

### Statistical analysis

Continuous variables were described using mean and standard deviation if they followed a normal distribution, or represented as median with interquartile range when the distribution deviated from normality. For categorical data, frequencies and their proportions were calculated. Group comparisons were made using statistical tests such as the Student's *t*-test, the Mann–Whitney *U*-test, or the chi-square test with Yates’ correction, depending on the specific conditions. Any variable showing significance at *P* < 0.05 in univariate analysis was incorporated into a multivariate logistic regression model to explore the association between platelet CD147 expression and the occurrence of ACS. The Benjamini–Hochberg method was implemented to adjust for multiple comparisons and control the false discovery rate at a cutoff of 0.05. Non-linear associations between CD147 expression and ACS risk were examined using restricted cubic spline models. A two-sided *P*-value below 0.05 was regarded as statistically significant. All analyses were conducted using R (version 4.2.0).

## Results

### Differential expression analysis

**Figure 2:**
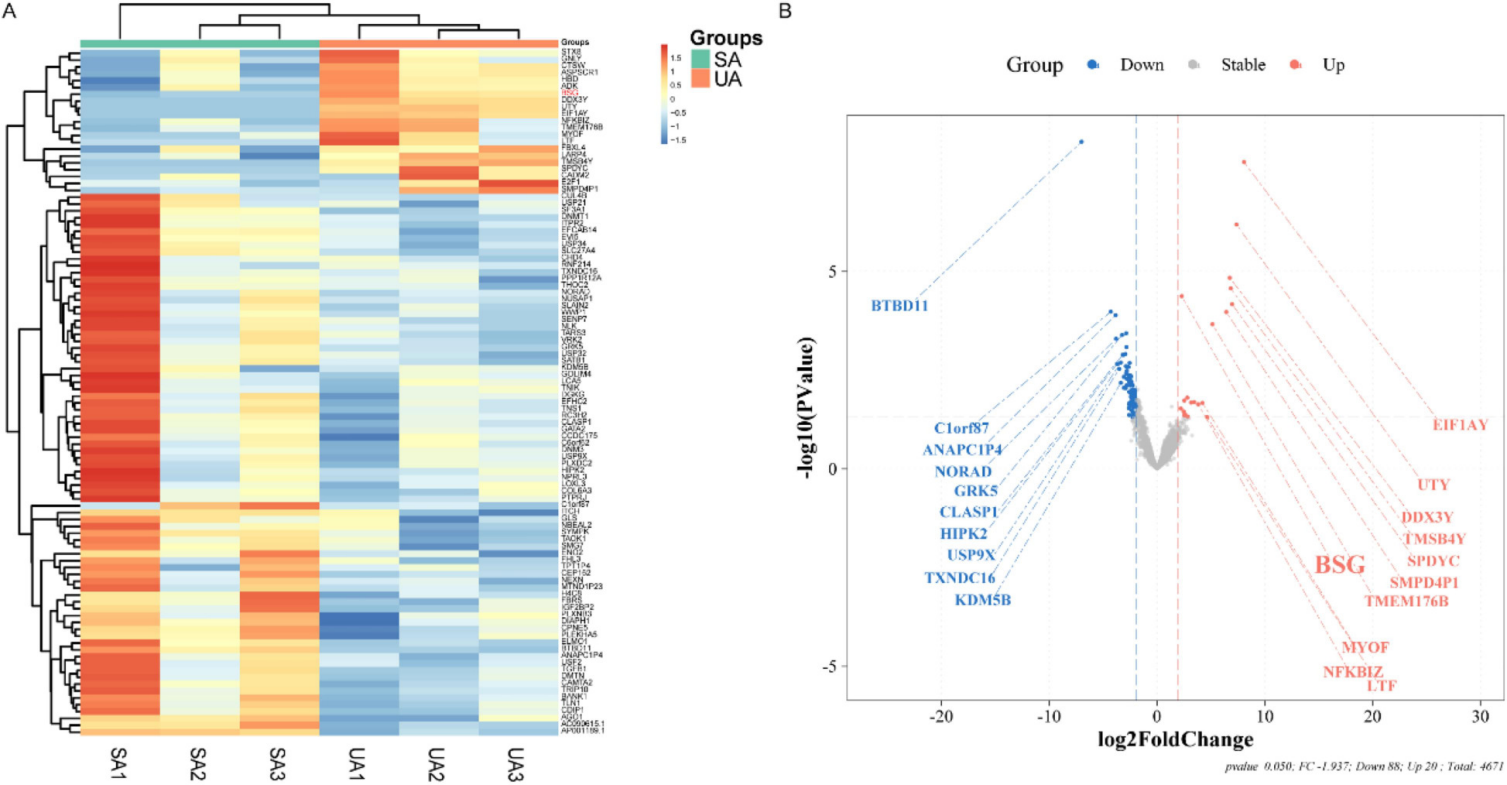
Exploratory and preliminary analysis of differential gene expression in platelets from ACS and SA patients (*n* = 3 per group). **(A)** Displays a heatmap with hierarchical clustering of differentially expressed genes (DEGs) between SA and UA groups. Each row represents a gene, and each column represents a patient sample, with color intensity reflecting the expression level, ranging from blue (low expression) to red (high expression). **(B)** Illustrates a volcano plot showing the log_2_ fold change versus the negative log_10_ of the *p*-value for each DEG. Points are color-coded as blue for downregulated, red for upregulated, and gray for no significant change in expression. Key genes with substantial fold changes and statistical significance are labeled, such as BSG with a marked upregulation in the UA group.

To identify the transcriptomic changes that characterize plaque instability, we performed a high-throughput sequencing analysis between the Unstable Angina (UA) and Stable Angina (SA) groups. This analysis revealed 108 differentially expressed genes (DEGs) that met a stringent significance threshold (fold change ≥4.7) (Figure 2). Of these genes, 20 were upregulated while a majority (88 genes) were downregulated in the UA group compared to the SA group. This result indicates that the progression to an unstable plaque phenotype is accompanied by a distinct and significant shift in the gene expression profile.

### Functional enrichment analysis

**Figure 3:**
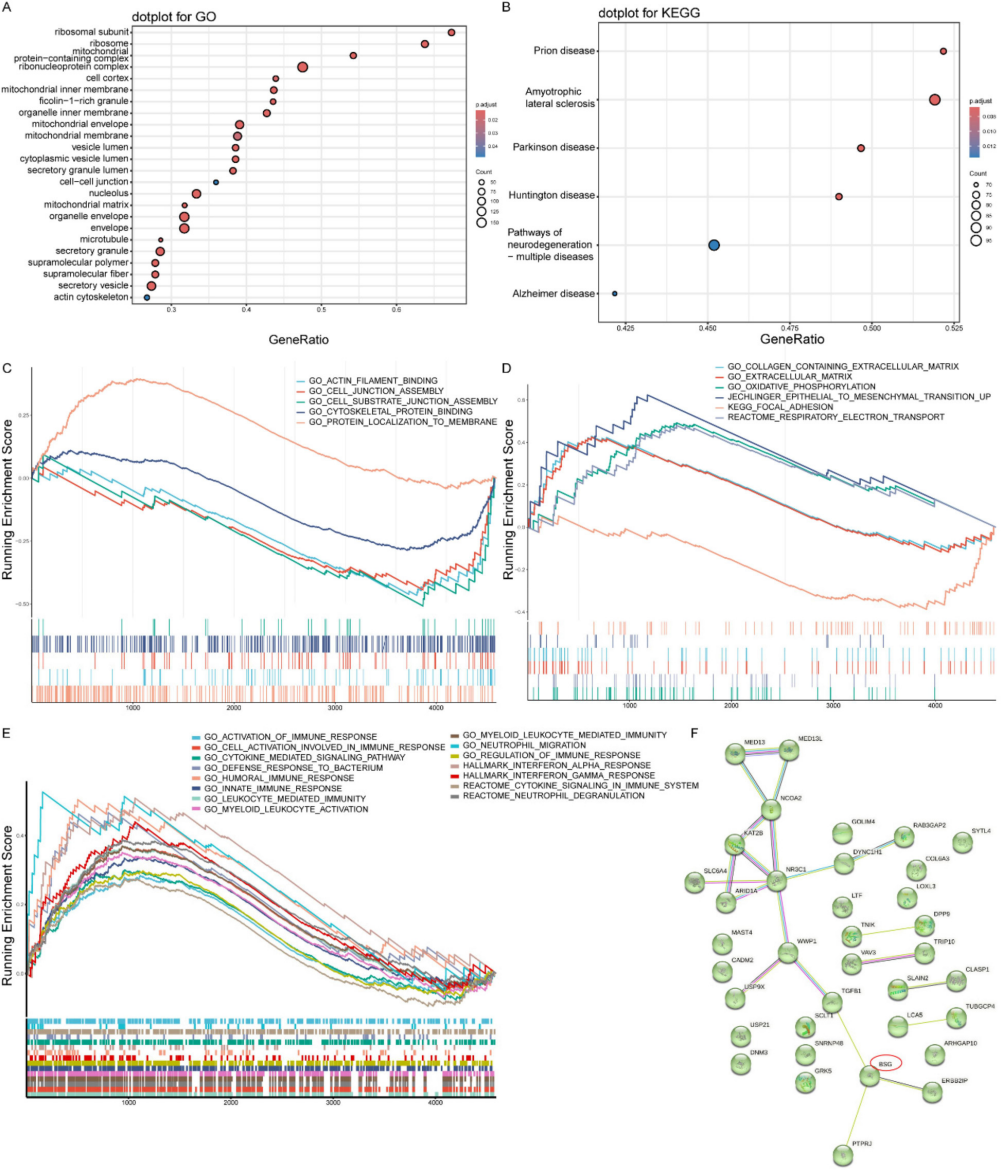
Functional enrichment analysis results. **(A)** GO enrichment analysis of cellular components. Dot size indicates gene count, and color represents adjusted *p*-value. **(B)** KEGG pathway enrichment analysis. Dot size indicates gene count, and color represents adjusted *p*-value. **(C)** Enrichment of CD147-associated functional pathways, demonstrating the coordinated regulation of our primary molecule of interest's network. **(D)** Enrichment of plaque stability-related gene sets, providing a mechanistic link from the platelet transcriptome to the clinical phenotype. **(E)** Enrichment of general inflammation and immune response pathways, confirming the overarching pathological context of ACS. **(F)** Protein-protein interaction network of differentially expressed genes constructed using STRING database.

To elucidate the biological functions of the identified DEGs, we performed GO enrichment analysis. Within the “Cellular Component” category, the analysis showed that the DEGs were significantly overrepresented in structures related to protein synthesis (e.g., “ribosomal subunit”) and energy metabolism (mitochondrial components) (Figure 3A). Furthermore, components associated with intracellular transport (“vesicles”) and cellular architecture (“cytoskeleton”) were also significantly enriched. These findings indicate that the unstable plaque phenotype is associated with a profound disruption of core cellular machinery responsible for protein production, energy homeostasis, and structural integrity.

To map the DEGs onto higher-level biological pathways, we conducted a KEGG pathway analysis. This analysis yielded a surprising result: the most significantly enriched pathways were related to neurodegenerative diseases, including Prion disease, Amyotrophic lateral sclerosis, and Parkinson's disease (Figure 3B). However, given the very small sample size of the discovery cohort, this unexpected finding should be interpreted with extreme caution, as it is highly likely to be a statistical artifact rather than a robust biological signal. It is reported here for completeness, but these speculative pathways do not form a basis for our study's main conclusions.

To investigate the functional status of specific biological concepts beyond individual gene lists, we employed Gene Set Enrichment Analysis (GSEA). This approach allowed us to test several hypotheses: First, to understand the role of our primary molecule of interest, we analyzed CD147-associated pathways. This revealed a significant negative enrichment in processes like “actin filament binding” and “cell-cell junction assembly” (Figure 3C and Supplementary Table S3), indicating that cellular functions directly related to CD147 are suppressed in the unstable condition. Second, to confirm the relevance to plaque biology, we assessed gene sets known to be involved in plaque stability. As expected, pathways such as “extracellular matrix organization”, “epithelial-to-mesenchymal transition”, and “focal adhesion” were significantly enriched (Figure 3D and Supplementary Table S3), confirming that extensive tissue remodeling and cell adhesion dynamics are active processes. Finally, to characterize the immune environment, we examined inflammation-related pathways. This showed a coordinated positive enrichment of multiple immune processes, including “myeloid leukocyte-mediated immunity”, “cytokine signaling”, and “neutrophil degranulation” (Figure 3E and Supplementary Table S3), highlighting a robust and active inflammatory response as a key feature of the disease state.

### PPI network

To investigate the functional interplay among the DEGs and to identify potential master regulators, we constructed a protein-protein interaction (PPI) network using the STRING database. The resulting network analysis revealed that CD147/BSG occupied a central position as a highly connected hub node (Figure 3F). Critically, its interacting partners were proteins directly involved in key pathological processes such as inflammation, cell adhesion, immune regulation, and angiogenesis. This finding suggests that CD147 may function as a central coordinator, orchestrating the diverse cellular events that contribute to the unstable plaque phenotype.

### Participants

The analysis included 180 patients (90 ACS, 90 SA) who met the eligibility criteria. Baseline characteristics are summarized in Table 1.

**Table 1:** Characteristics of stable angina and acute coronary syndrome.

Variables	ACS	SA	*p* Value	Reference range
	*N* = 90	*N* = 90		
Gender			0.880	–
Male	52 (57.8%)	50 (55.6%)		
Female	38 (42.2%)	40 (44.4%)		–
Age (year)	55.0 [48.2;66.5]	70.0 [65.0;75.0]	<0.001	–
BMI (kg/m^2^)	25.2 (2.87)	22.4 (2.95)	<0.001	–
Hypertension			0.232	–
No	46 (51.1%)	37 (41.1%)		–
Yes	44 (48.9%)	53 (58.9%)		–
Diabetes			0.761	–
No	55 (61.1%)	52 (57.8%)		–
Yes	35 (38.9%)	38 (42.2%)		–
Smoking status			0.002	–
No	42 (46.7%)	48 (53.3%)		–
Quitted	16 (17.8%)	29 (32.2%)		–
Current	32 (35.6%)	13 (14.4%)		–
Platelet (*10^9^/L)	262 [213;338]	258 [206;312]	0.276	125–350
PDW (%)	15.4 [12.9;16.6]	13.6 [11.8;15.8]	0.002	9–17
PCT (%)	0.22 [0.18;0.25]	0.20 [0.17;0.25]	0.137	0.11∼0.27
BNP (pg/ml)	108 [48.3;158]	53.2 [33.4;78.9]	<0.001	0∼100
cTnI (ng/L)	9,097[3,857;13,847]	17.6 [6.82;38.2]	<0.001	<54
TC (mmol/L)	4.94 [4.29;5.54]	4.21 [3.52;5.18]	<0.001	0–5.2
TG (mmol/L)	1.94 [1.31;2.51]	1.46 [1.01;1.83]	<0.001	0–1.7
HDL (mmol/L)	1.35 (0.36)	1.32 (0.34)	0.618	1.04–1.66
LDL (mmol/L)	2.91 [2.42;3.31]	2.06 [1.50;2.50]	<0.001	0–3.12
CD147_platelet	1.07 [0.89;1.27]	0.91 [0.70;1.10]	<0.001	–

Continuous data conforming to a normal distribution were reported as mean (SD) and those not conforming as median (quartiles). Statistical significance was determined using Student's *t*-test, Rank-sum test or *χ*² test, with *p*-values calculated. ACS, acute coronary syndrome; BMI, body mass index; BNP, brain natriuretic peptid; CD147_platelet, sample OD readings/standard control readings; cTnI, cardiac troponin I; PDW, platelet distribution width; PCT, plateletcrit; SA, stable angina; TC, total cholesterol; TG, triglyceride; HDL, high density lipoprotein; LDL, low density lipoprotein.

### Descriptive data

Between ACS and SA groups, significant differences (*P* < 0.05) were found in age, BMI, smoking status, PDW, BNP, cTnI, total cholesterol, triglycerides, and LDL. Gender, hypertension, platelet count, plateletcrit, and HDL were comparable between groups (Table 1).

### Flow cytometry analysis

To validate our transcriptomic findings at the protein level, we quantified the surface expression of CD147 on platelets from both patient cohorts using flow cytometry. The analysis revealed that platelet CD147 expression was significantly elevated in the ACS group [median: 1.07 (IQR: 0.89–1.27)] compared to the SA group [median: 0.91 (IQR: 0.70–1.10)] (*P* < 0.001) (Table 1, Supplementary Figures S1, S2). This result confirms that the increased gene expression of CD147 in the ACS population translates to a higher protein level on the surface of circulating platelets, directly linking this molecule to the acute phase of coronary artery disease.

### Logistic regression analysis

To determine if the association between platelet CD147 and ACS was independent of established clinical risk factors, we conducted a multivariable logistic regression analysis. After adjusting for a comprehensive panel of traditional cardiovascular risk factors—including age, gender, BMI, smoking status, hypertension, diabetes, and LDL-C—platelet CD147 expression remained a strong and statistically significant predictor of ACS (adjusted OR = 9.21, 95% CI: 2.33–36.42, *P* = 0.002) (Table 2). This finding indicates that platelet CD147 provides additional diagnostic value beyond that of conventional risk markers, highlighting its potential as a novel, independent biomarker for ACS.

**Table 2:** Univariate and multivariate logistic regression analysis of traditional coronary heart disease risk factors and platelet surface CD147 expression between stable angina (SA) and acute coronary syndrome (ACS).

Variable	OR (95% CI)	*p* Value	OR (95% CI). multi	*p* Value. multi
Age	0.87 (0.83–0.90)	*p* < .001	0.89 (0.84–0.93)	*p* < .001
BMI	1.39 (1.23–1.57)	*p* < .001	1.18 (1.01–1.38)	*p* = .034
Gender				
Male				
Female	0.91 (0.51–1.65)	*p* = .764		
Hypertension				
No				
Yes	0.67 (0.37–1.20)	*p* = .179	0.75 (0.32–1.77)	*p* = .519
Diabetes				
No				
Yes	0.87 (0.48–1.58)	*p* = .649		
LDL	4.43 (2.69–7.30)	*p* < .001	2.63 (1.43–4.81)	*p* = .002
Smoking status				
No	0.63 (0.30–1.32)	*p* = .220	0.52 (0.20–1.38)	*p* = .188
Quitted	2.81 (1.31–6.05)	*p* = .008	1.26 (0.40–4.00)	*p* = .691
Current				
CD147 platelet	5.44 (2.00–14.84)	*p* < .001	9.21 (2.33–36.42)	*p* = .002

OR, odds ratio; CI, confidence interval.

### Non-linear association of Cd147 expression and ACS risk

**Figure 4:**
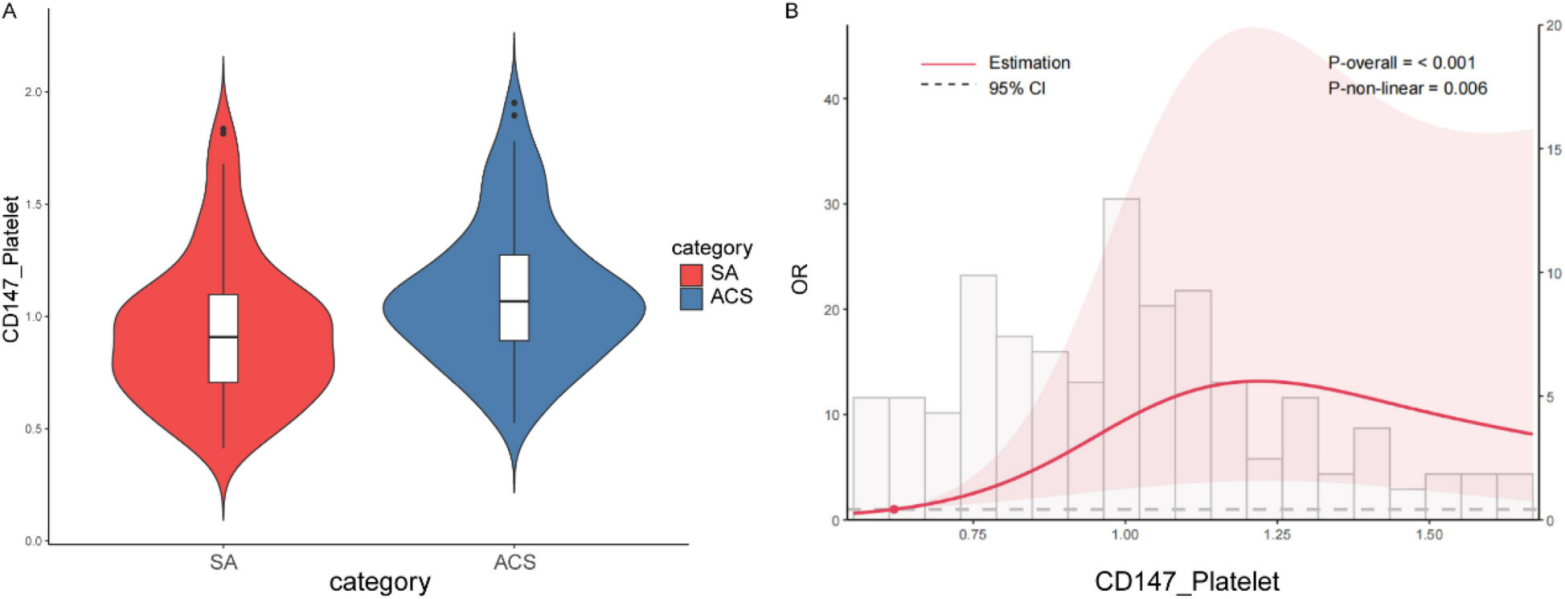
Cd147 expression and ACS risk. **(A)** Violin plot comparing the distribution of CD147 expression levels on platelets between patients with Stable Angina (SA) and Acute Coronary Syndrome (ACS). The ACS group exhibits generally higher CD147 expression levels compared to the SA group. **(B)** Restricted cubic spline plot showing the non-linear relationship between CD147 expression and the odds ratio (OR) of developing ACS. The overall model is significant (*P*-overall < 0.001), with a notable non-linear relationship (*P*-non-linear = 0.006). Initially, the risk of ACS increases sharply with higher CD147 expression but plateaus around a CD147 expression level of 1.2.

To further explore the precise nature of the association between platelet CD147 and ACS, we performed a restricted cubic spline (RCS) analysis. This model confirmed that CD147 expression is a highly significant predictor of ACS overall (*P*-overall < 0.001) but revealed that the relationship is significantly non-linear (*P*-non-linear = 0.006). As illustrated in Figure 4, the dose-response curve shows that the risk of ACS escalates sharply as CD147 expression increases from low to moderate levels. However, this risk does not increase indefinitely; it appears to plateau after reaching a threshold of approximately 1.2. This finding suggests that the pro-thrombotic or inflammatory impact of CD147 may exert its maximum effect at this level, uncovering a more complex biological relationship than a simple linear model would suggest.

## Discussion

We investigated platelet CD147 expression differences between SA and ACS patients, and its relationship with coronary plaque stability. High-throughput sequencing identified 108 DEGs, with CD147 emerging as a hub node in cardiovascular-related biological processes. Flow cytometry confirmed significantly higher platelet CD147 expression in ACS compared to SA patients (*P* < 0.001), consistent with sequencing results. Multivariable logistic regression revealed a strong association between platelet CD147 expression and plaque instability (adjusted OR = 9.21, 95% CI: 2.33–36.42, *P* = 0.002). While the width of this confidence interval reflects the statistical imprecision expected in an exploratory study with a modest sample size, it is critical to highlight that the lower bound of the CI is 2.33. This indicates that, even under the most conservative estimate, high CD147 expression is associated with at least a 133% increase in the odds of ACS, a clinically meaningful effect. Crucially, this association persisted after adjusting for well-established cardiovascular risk factors, including LDL-C and smoking status, underscoring that platelet CD147 provides independent predictive information beyond these traditional markers. These findings strongly suggest that platelet CD147 is a promising candidate for regulating plaque stability, warranting further mechanistic validation.

Functional enrichment analyses provided comprehensive insights into the molecular mechanisms of CD147 under moderate altitude conditions. GO analysis highlighted the enrichment of cellular components related to ribosomal and mitochondrial structures, indicating CD147's potential involvement in protein synthesis and energy metabolism regulation. GSEA further revealed negative enrichment of pathways related to actin filament binding and cell-cell junction assembly, implying CD147's role in regulating cellular structural stability and intercellular interactions. Additionally, the enrichment of plaque stability-related pathways and multiple inflammation-related processes suggests that CD147 may be critically involved in vascular remodeling and inflammatory responses (23, 24).

Protein–protein interaction (PPI) analysis further indicated that CD147 is integrally involved in key biological processes, including inflammatory response, cell adhesion, immune regulation, and angiogenesis. These results align with previous studies, supporting the notion that CD147's upregulation in unstable plaques is linked to its role in promoting inflammation and thrombus formation. This indicates that CD147 not only serves as a marker of heightened inflammation and thrombosis in ACS but may also play an active role in plaque destabilization (8, 25). CD147 induces matrix metalloproteinase (MMP) secretion, promoting plaque instability through fibrous cap degradation (26). Under moderate altitude conditions, hypoxia amplifies plaque vulnerability by upregulating CD147 and enhancing inflammatory-thrombotic cascades. This highlights the importance of considering geographic and environmental influences when evaluating molecular mechanisms in ACS. These findings suggest that elevated platelet CD147 may not only reflect the intensified inflammatory and thrombotic activity in ACS but also actively contribute to the instability of atherosclerotic plaques. This positions CD147 as a critical risk factor and potential prognostic marker for ACS.

A key finding of our study is the significant non-linear association between platelet CD147 expression and ACS risk, as demonstrated by restricted cubic spline analysis (*P* for non-linearity = 0.006). This relationship exhibits a clear threshold effect, suggesting that platelet CD147 acts as a biological “switch” rather than a linear risk factor. We postulate that below a certain expression threshold, its pathological effects are controlled by endogenous regulatory mechanisms. However, once expression surpasses this critical tipping point, it likely triggers an uncontrolled, self-amplifying cascade of pro-inflammatory and pro-thrombotic events, such as massive MMP release and enhanced platelet aggregation, leading to a sharp increase in ACS risk. The subsequent plateau at very high expression levels may indicate a “ceiling effect”, where these pathological pathways have become saturated. This threshold-dependent behavior underscores the potential of platelet CD147 as a marker for identifying individuals who have transitioned into a high-risk state for plaque rupture.

Future research should focus on how CD147 influences platelet activation and thrombus formation via different biological pathways, thereby affecting plaque stability. Furthermore, these observations identify CD147 as a therapeutic target for coronary heart disease. Targeting CD147 to inhibit its expression or function could be a novel approach to stabilize vulnerable plaques, reduce inflammation, and prevent acute coronary events. In particular, therapeutic strategies aimed at mitigating the effects of chronic hypoxia and other high-altitude environmental stressors on CD147 expression could offer tailored interventions for populations residing in moderate altitude regions. As a therapeutic target, CD147 presents a promising avenue for the development of new treatments aimed at improving cardiovascular outcomes.

## Conclusion

This study demonstrated that platelet CD147 expression serves as a highly promising predictor for differentiating plaque stability status in CHD under moderate altitude conditions. Our findings revealed that CD147 not only contributes to platelet activation and thrombus formation but may also directly influence plaque stability through multiple molecular pathways. The impact of chronic hypoxia and other environmental stressors in moderate altitude regions on CD147 expression provides new insights for risk stratification and targeted therapeutic strategies in altitude-specific populations.

However, several limitations should be acknowledged in our study. First, the critically small sample size (*n* = 3 vs. 3) for the initial high-throughput sequencing means that the resulting list of 108 DEGs and the associated exploratory bioinformatics analyses (e.g., GO/KEGG) should be viewed as hypothesis-generating only and are susceptible to false positives. Second, the wide 95% confidence interval (2.33–36.42) for the odds ratio of CD147 is a direct consequence of our study's modest sample size, a common feature of initial, exploratory investigations. However, it is essential to interpret this result with nuance. Despite the imprecision, the association is highly statistically significant (*p* = 0.002), strongly suggesting the finding is not due to chance. Most importantly, the CI's lower bound of 2.33 represents a substantial and clinically relevant effect size, indicating a robust association even at its most conservative estimate. Therefore, rather than invalidating our conclusion, this finding underscores the urgent need for validation in larger, prospective, multicenter cohorts to obtain a more precise estimate of CD147's true effect size. Third, the absence of longitudinal data further restricts our ability to assess temporal changes in CD147 expression and its long-term clinical implications. Fourth, our study groups exhibited significant differences in baseline characteristics, most notably age. Although our multivariate analysis statistically controlled for these variables, the large discrepancy could mask residual confounding from unmeasured age-related biological factors. Future studies, perhaps using age-matched cohorts, would be beneficial to corroborate these findings.

Future research should address these limitations by expanding the cohort size to improve statistical precision, incorporating long-term follow-up, and further investigating the regulatory mechanisms of CD147. Additionally, studies should examine how altitude-related factors modulate CD147 expression in cardiovascular risk assessment.

## Data Availability

The original contributions presented in the study are publicly available. Raw data have been deposited to National Center for Biotechnology Information (NCBI) under the BioProject number PRJNA1337309.
