# Supplementary material for: Differential expression of platelet CD147 in moderate altitude conditions: implications for coronary plaque stability assessment
Source: Front Cardiovasc Med. 2025 Oct 27;12:1621491. doi: 10.3389/fcvm.2025.1621491 (PMC12598010; doi:10.3389/fcvm.2025.1621491)
Supplement: Supplementary file 1 [file Datasheet1.pdf]

TableS1. Differential Expressed Genes between Stable Angina (SA) and Unstable Angina by High-Throughput Sequencing.

Symbol	baseMean	log2FoldChange	lfcSE	stat	pvalue	padj	group
BTBD11	46.6487401	-6.999870754	1.198403917	-5.840994555	5.19E-09	2.42E-05	Down
C1orf87	34.7626839	-4.291814255	1.106770371	-3.877782029	0.000105413	0.055494416	Down
ANAPC1P4	114.8821251	-3.842626767	1.003584003	-3.828903966	0.000128715	0.060122878	Down
AP001189.1	28.97178727	-2.865261302	0.804455042	-3.56174199	0.000368402	0.143400608	Down
GRK5	369.4252715	-3.246005477	0.918461446	-3.534177171	0.000409047	0.146973657	Down
NORAD	91.19381177	-3.800712359	1.093669536	-3.475192673	0.000510487	0.170320192	Down
SATB1	201.607794	-2.83267845	0.847020612	-3.344285145	0.000824949	0.256889119	Down
TGFB1	2322.045551	-2.935747733	0.909160043	-3.229076942	0.001241905	0.339891555	Down
SENP7	233.8358911	-3.120350623	0.970914867	-3.213825155	0.001309794	0.339891555	Down
CLASP1	198.2166711	-3.181495203	0.9890049	-3.216864955	0.001295996	0.339891555	Down
USP9X	1839.725616	-3.365426414	1.091213388	-3.084113934	0.002041594	0.487674932	Down
SYMPK	37.84950176	-2.546876067	0.827604431	-3.07740748	0.002088096	0.487674932	Down
HIPK2	176.9890083	-3.705910544	1.210514302	-3.061434745	0.00220279	0.489963435	Down
SLAIN2	433.2414809	-2.892930428	0.957436343	-3.02153814	0.00251494	0.533967393	Down
TLN1	19649.7356	-2.654176064	0.882477714	-3.007640899	0.00263284	0.534695552	Down
TXNDC16	46.14244838	-3.504650033	1.178383316	-2.974117153	0.002938329	0.57187222	Down
NLK	135.3814398	-2.803492383	0.954736643	-2.936403881	0.003320417	0.620386719	Down
WWP1	489.7739319	-2.543315073	0.895245944	-2.840912142	0.00449847	0.685231105	Down

USF2	182.4555628	-2.48560589	0.870714284	-2.854674529	0.004308095	0.685231105	Down
TNS1	252.9690264	-2.453026501	0.870663098	-2.817423303	0.004841068	0.685231105	Down
PTPRJ	398.2189809	-2.87571955	0.995686076	-2.888178934	0.003874794	0.685231105	Down
MTND1P23	165.6711222	-2.418074741	0.85255979	-2.836252388	0.004564636	0.685231105	Down
H4C8	59.36151955	-2.455549812	0.869999157	-2.822473784	0.004765472	0.685231105	Down
DMTN	441.2706327	-2.613693419	0.913589826	-2.860904691	0.00422434	0.685231105	Down
C6orf62	2285.061472	-3.084955659	1.09179333	-2.825585736	0.004719426	0.685231105	Down
NUSAP1	48.74096354	-2.808990952	1.005433111	-2.793811863	0.005209077	0.702772782	Down
EFHC2	474.3357474	-2.538813753	0.909870926	-2.790300997	0.005265906	0.702772782	Down
SLC27A4	32.83491883	-2.471713689	0.891768155	-2.77169988	0.005576442	0.723543406	Down
CDIP1	44.53531817	-2.6248149	0.956712723	-2.743576871	0.006077382	0.767228378	Down
ITPR2	257.7938167	-2.357087827	0.863494216	-2.729708877	0.006339028	0.77919996	Down
KDM5B	99.74570704	-3.359023905	1.237584832	-2.714176691	0.006644072	0.795755375	Down
NBEAL2	251.6601108	-2.461021351	0.924245581	-2.662735318	0.007750835	0.84195703	Down
KAT2B	88.95708	-2.245999007	0.843091693	-2.664003246	0.007721682	0.84195703	Down
GATA2	74.34976409	-2.820341728	1.057578564	-2.666791691	0.007657912	0.84195703	Down
EVI5	31.59258533	-2.572177588	0.962708368	-2.67181389	0.007544248	0.84195703	Down
COL6A3	51.25118148	-3.083652466	1.17887426	-2.615760281	0.008902904	0.936652197	Down
CCDC175	464.2044143	-2.900295938	1.11073177	-2.611157812	0.009023624	0.936652197	Down
TRIP10	38.86865623	-2.387570816	0.931102138	-2.564241577	0.010340155	0.985691079	Down

FBXW7	56.62182165	-2.197097726	0.852062749	-2.578563292	0.009921212	0.985691079	Down
ALDOA	6716.142356	-2.070259437	0.805969814	-2.568656294	0.010209366	0.985691079	Down
ZZEF1	53.80777854	-2.098254181	0.90262582	-2.3246113	0.020092754	0.997784287	Down
VAV3	101.6807328	-1.942933732	0.821314836	-2.365638178	0.017999025	0.997784287	Down
USP21	26.44310033	-2.325992072	1.09011317	-2.133716145	0.032866024	0.997784287	Down
TUBGCP4	131.3856189	-2.062569228	0.826874567	-2.494416095	0.012616455	0.997784287	Down
TPT1P4	31.01737813	-2.556137336	1.111844282	-2.299006594	0.021504565	0.997784287	Down
TNIK	343.9635329	-2.454177464	1.065836548	-2.302583325	0.021302298	0.997784287	Down
SYTL4	263.0760677	-2.195637483	0.96606115	-2.272772778	0.023039876	0.997784287	Down
SNRNP48	48.27317944	-2.020686787	1.002058694	-2.016535357	0.043744025	0.997784287	Down
SLC6A4	54.55060478	-2.040709818	0.98487574	-2.072048011	0.038260964	0.997784287	Down
SCLT1	588.4741176	-2.048048144	0.805884894	-2.541365597	0.011042039	0.997784287	Down
RSRC1	111.5416223	-1.992408639	0.898366325	-2.21781314	0.026567576	0.997784287	Down
RNF214	46.28195047	-2.344671905	1.100686086	-2.130191283	0.033155824	0.997784287	Down
RANBP9	47.17414699	-2.151046512	1.050645705	-2.047356688	0.040623076	0.997784287	Down
RAB3GAP2	76.42528365	-2.141585242	0.930840609	-2.300700272	0.021408579	0.997784287	Down
PTPN12	1623.207019	-2.044329816	0.841840223	-2.428405961	0.015165357	0.997784287	Down
PLXNB3	74.1944385	-2.629031818	1.205920498	-2.180103766	0.029249771	0.997784287	Down
PLXDC2	1055.828174	-2.579306007	1.017532065	-2.534864597	0.01124908	0.997784287	Down
PLEKHA5	44.48434832	-2.327739655	0.991469579	-2.347767096	0.018886327	0.997784287	Down

PIAS4	41.61312294	-2.177855389	0.897662122	-2.426141568	0.015260316	0.997784287	Down
PDE3A	43.6730479	-2.297698768	1.151194752	-1.995925332	0.045942051	0.997784287	Down
NSD2	35.64870182	-2.100449657	0.944594238	-2.223652837	0.026171811	0.997784287	Down
NPRL3	169.1087297	-2.424451387	0.988988506	-2.451445463	0.014228375	0.997784287	Down
NLRC5	141.2507382	-2.065237502	0.863565816	-2.39152299	0.016778633	0.997784287	Down
NCOA2	105.858099	-2.295936431	0.919632599	-2.496580083	0.012539735	0.997784287	Down
NBPF3	25.57667692	-2.164238104	0.953516249	-2.269744334	0.0232231	0.997784287	Down
NBAS	263.0656198	-2.213576173	0.945517014	-2.341127807	0.019225584	0.997784287	Down
MED13L	45.47775867	-2.227052406	0.993280142	-2.242119129	0.024953674	0.997784287	Down
MED13	33.2359403	-2.062268416	0.862404531	-2.391300533	0.016788804	0.997784287	Down
MAST4	129.3251944	-2.188614378	0.958060921	-2.284420886	0.022346807	0.997784287	Down
MAP7D3	45.68426272	-2.0573772	1.019180885	-2.018657562	0.043522824	0.997784287	Down
LOXL3	162.7257358	-2.384602178	1.061387439	-2.246684	0.024660229	0.997784287	Down
LCA5	33.70412506	-2.585960579	1.279669411	-2.020803621	0.043300098	0.997784287	Down
KPNA5	81.00145228	-2.259469596	0.941670563	-2.399426812	0.016420762	0.997784287	Down
HECTD4	95.728575	-2.094820216	0.878454711	-2.384665013	0.01709468	0.997784287	Down
GOLIM4	32.37525697	-2.598549669	1.145555276	-2.268375628	0.023306322	0.997784287	Down
ERBIN	614.9111166	-2.087630705	0.938012026	-2.225590555	0.026041619	0.997784287	Down
DYNC1H1	136.4686436	-2.200167432	0.998498856	-2.203475164	0.027561276	0.997784287	Down
DPP9	33.62070948	-1.955458343	0.86486951	-2.260986566	0.023760089	0.997784287	Down

DNM3	805.9890793	-2.401224773	0.979679413	-2.451031165	0.014244762	0.997784287	Down
DCAF11	38.05584946	-1.939814923	0.876309309	-2.213618984	0.026855	0.997784287	Down
CPEB4	57.29995073	-2.16748356	0.960235701	-2.257241172	0.023993009	0.997784287	Down
CDKL1	165.5488704	-2.257338838	1.14988371	-1.96310185	0.049634341	0.997784287	Down
BAZ2A	94.12793864	-2.26036235	0.945162171	-2.391507426	0.016779344	0.997784287	Down
ARID1A	37.82158847	-2.084854289	0.843360546	-2.472079466	0.013432965	0.997784287	Down
ARHGAP10	116.2023828	-2.23176023	0.908876519	-2.455515335	0.014068275	0.997784287	Down
ANKIB1	34.59556427	-2.217943941	1.068442222	-2.075866991	0.037906254	0.997784287	Down
AGPAT1	39.02489954	-2.15616031	0.957765559	-2.251240182	0.024370329	0.997784287	Down
AGO1	34.71790753	-2.41834257	0.999143826	-2.420414866	0.015502809	0.997784287	Down
EIF1AY	42.70537303	8.062088088	1.429378644	5.640274621	1.70E-08	3.97E-05	Up
UTY	26.38353727	7.367923269	1.481086003	4.974676185	6.54E-07	0.001017605	Up
DDX3Y	17.18302312	6.74812723	1.556715892	4.334848296	1.46E-05	0.01703288	Up
TMSB4Y	18.33211892	6.844129787	1.630459262	4.197669911	2.70E-05	0.025193068	Up
BSG	548.5468378	2.297918019	0.561401682	4.093179789	4.25E-05	0.033124983	Up
SPDYC	19.98231926	6.968411396	1.749186517	3.983801229	6.78E-05	0.045256389	Up
SMPD4P1	46.09426906	6.427969736	1.659124797	3.874313582	0.000106926	0.055494416	Up
TMEM176B	36.23628703	5.148340316	1.392570627	3.697004816	0.000218158	0.092637907	Up
STX8	22.60147207	2.467013367	1.17178554	2.105345461	0.035261248	0.997784287	Up
NFKBIZ	11.098643	3.82126903	1.683941738	2.269240642	0.023253696	0.997784287	Up

MYOF	10.35209142	4.211552303	1.832058808	2.298808469	0.021515818	0.997784287	Up
LTF	3.993550439	4.639272996	2.357873104	1.967566867	0.049117895	0.997784287	Up
LARP4	20.31625603	2.206031992	1.014250524	2.17503658	0.029627375	0.997784287	Up
HBD	11.51110954	2.543328198	1.266519707	2.008123666	0.044630152	0.997784287	Up
GNLY	297.0354069	2.805735976	1.163482942	2.411497302	0.015887169	0.997784287	Up
FBXL4	29.57222947	2.834729648	1.428816597	1.983970269	0.047259154	0.997784287	Up
E2F1	52.55857288	2.52713032	1.075116338	2.350564521	0.018744958	0.997784287	Up
CADM2	29.59700043	3.15697267	1.368311734	2.307202805	0.021043517	0.997784287	Up
ASPSCR1	11.57849187	3.443999499	1.484895182	2.319355293	0.02037578	0.997784287	Up
ADK	23.97699634	2.568661199	1.257754308	2.04225991	0.041125757	0.997784287	Up

TableS 2. Characteristics of Stable Angina (SA) and Acute Coronary Syndrome(ACS).

	**ACS** *N=90*	**SA** *N=90*	**p.overall**
Gender			0.880
male	52 (57.8%)	50 (55.6%)	
female	38 (42.2%)	40 (44.4%)	
Age(year)	55.0 [48.2;66.5]	70.0 [65.0;75.0]	<0.001
BMI (kg/m^2)	25.2 (2.87)	22.4 (2.95)	<0.001
Hypertension			0.232
No	46 (51.1%)	37 (41.1%)	
Yes	44 (48.9%)	53 (58.9%)	
Diabetes			0.761
No	55 (61.1%)	52 (57.8%)	
Yes	35 (38.9%)	38 (42.2%)	

	ACS<br>*N=90*	SA<br>*N=90*	p.overall
Smoking_status:			0.002
No	42 (46.7%)	48 (53.3%)	
quitted	16 (17.8%)	29 (32.2%)	
current	32 (35.6%)	13 (14.4%)	
WBC(*10^9/L)	10.4 [7.84;13.0]	6.66 [5.51;8.58]	<0.001
N（%）	77.6 [67.6;87.0]	56.2 [48.5;62.8]	<0.001
Mono.per(%)	6.29 (2.80)	6.20 (2.22)	0.803
Mono(*10^9/L)	0.57 [0.42;0.79]	0.44 [0.32;0.58]	<0.001
Platelet(*10^9/L)	262 [213;338]	258 [206;312]	0.276
PDW(fL)	15.4 [12.9;16.6]	13.6 [11.8;15.8]	0.002
MPV(fL)	10.0 (1.15)	9.56 (1.36)	0.020
PCT(ng/ml)	0.22 [0.18;0.25]	0.20 [0.17;0.25]	0.137
P-LCR (%)	13.9 [10.6;20.1]	13.1 [9.75;22.0]	0.493
GLU (mmol/L)	5.66 [4.90;6.40]	4.94 [4.33;5.52]	<0.001
UA(μmol/L)	348 [258;398]	324 [262;381]	0.209
BNP (pg/ml)	108 [48.3;158]	53.2 [33.4;78.9]	<0.001
cTn(ng/L)	9097 [3857;13847]	17.6 [6.82;38.2]	<0.001
CK(U/L)	654 [378;900]	99.0 [72.0;128]	<0.001
CK-MB(U/L)	57.0 [39.2;86.2]	14.0 [10.0;21.0]	<0.001
Cr(μmol/L)	92.6 (24.5)	80.8 (17.5)	<0.001
HCY(μmol/L)	13.7 [9.72;16.5]	9.84 [7.58;13.1]	<0.001
CRP (mg/L)	4.75 [2.60;8.20]	2.40 [1.02;3.88]	<0.001

	ACS<br>*N=90*	SA<br>*N=90*	p.overall
TC (mmol/L)	4.94 [4.29;5.54]	4.21 [3.52;5.18]	<0.001
TG (mmol/L)	1.94 [1.31;2.51]	1.46 [1.01;1.83]	<0.001
HDL (mmol/L)	1.35 (0.36)	1.32 (0.34)	0.618
LDL (mmol/L)	2.91 [2.42;3.31]	2.06 [1.50;2.50]	<0.001
TBIL(μmol/L)	14.6 [9.32;18.7]	11.1 [7.75;16.3]	0.001
IBIL(μmol/L)	9.80 [6.53;13.6]	7.80 [5.90;10.2]	0.001
DBIL(μmol/L)	3.70 [2.52;5.80]	2.60 [2.10;3.80]	<0.001
CD147_Platelet	1.07 [0.89;1.27]	0.91 [0.70;1.10]	<0.001

Continuous data conforming to a normal distribution were reported as mean (SD) and those not conforming as median(quartiles). Statistical significance was determined using t-test, Rank-sum test or χ^2 test, with p-values calculated.

SA: Stable Angina. ACS: Acute Coronary Syndrome. BMI: body mass index. WBC: white blood cell. N: neutrophil. Mono.Per: monocytes percentage. mono: monocytes. PDW: platelet distribution width. MPV: Mean Platelet Volume. PCT: procalcitonin. P-LCR: platelet -larger cell ratio. GLU: Glucose. UA: uric acid. cTn: cardiac troponin. CK: creatine kinase. CK-MB: creatine kinase-MB. Cr: creatinine. HCY: homocysteine. CRP: C-reactive protein. TC: total cholesterol. TG: Triglycerides. HDL: high-density lipoprotein. LDL: low-density lipoprotein. TBIL: total bilirubin. IBIL: indirect bilirubin. DBIL: direct bilirubin.

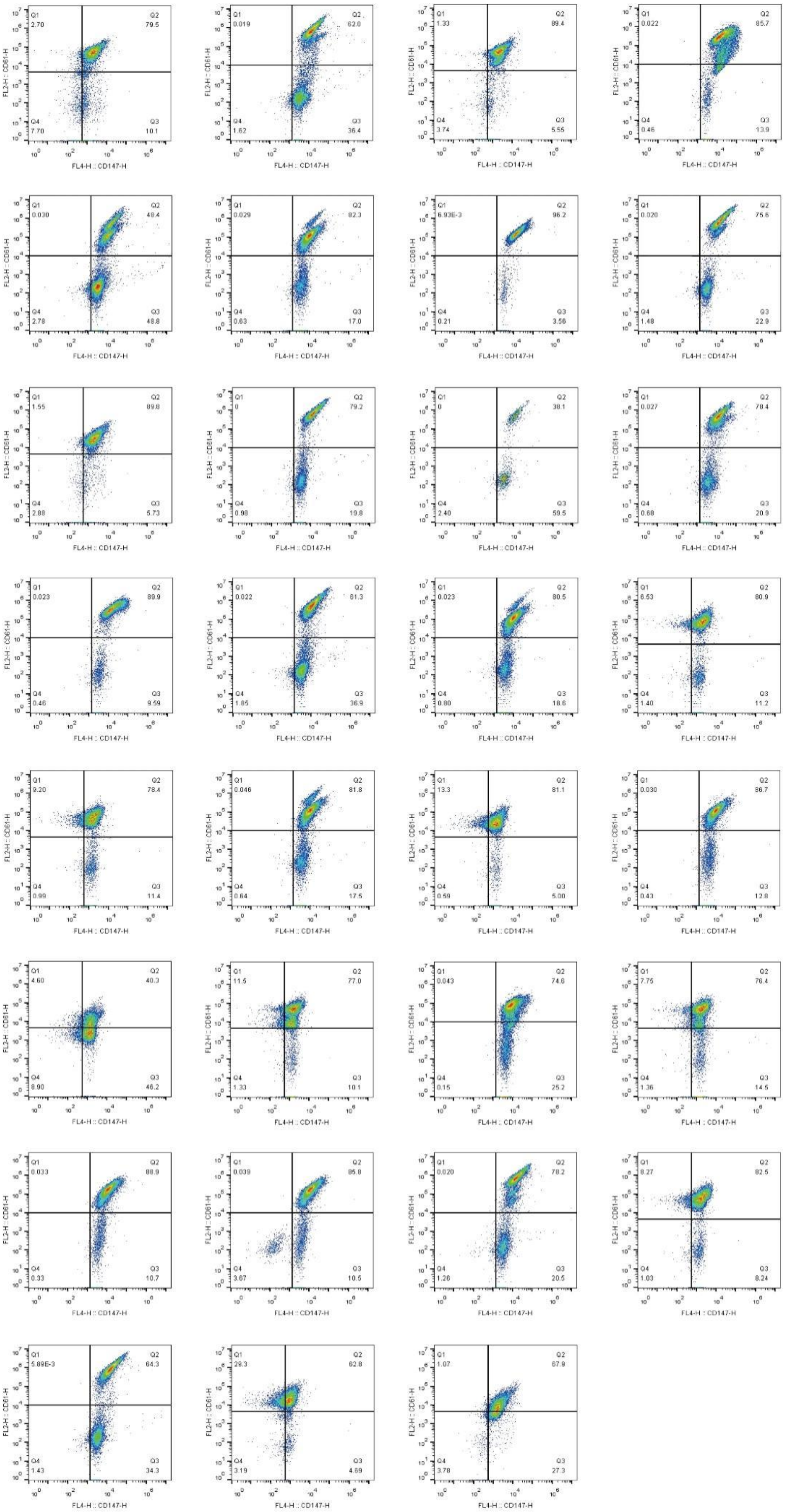
FL2-H :: CD61-H
FL4-H :: CD147-H

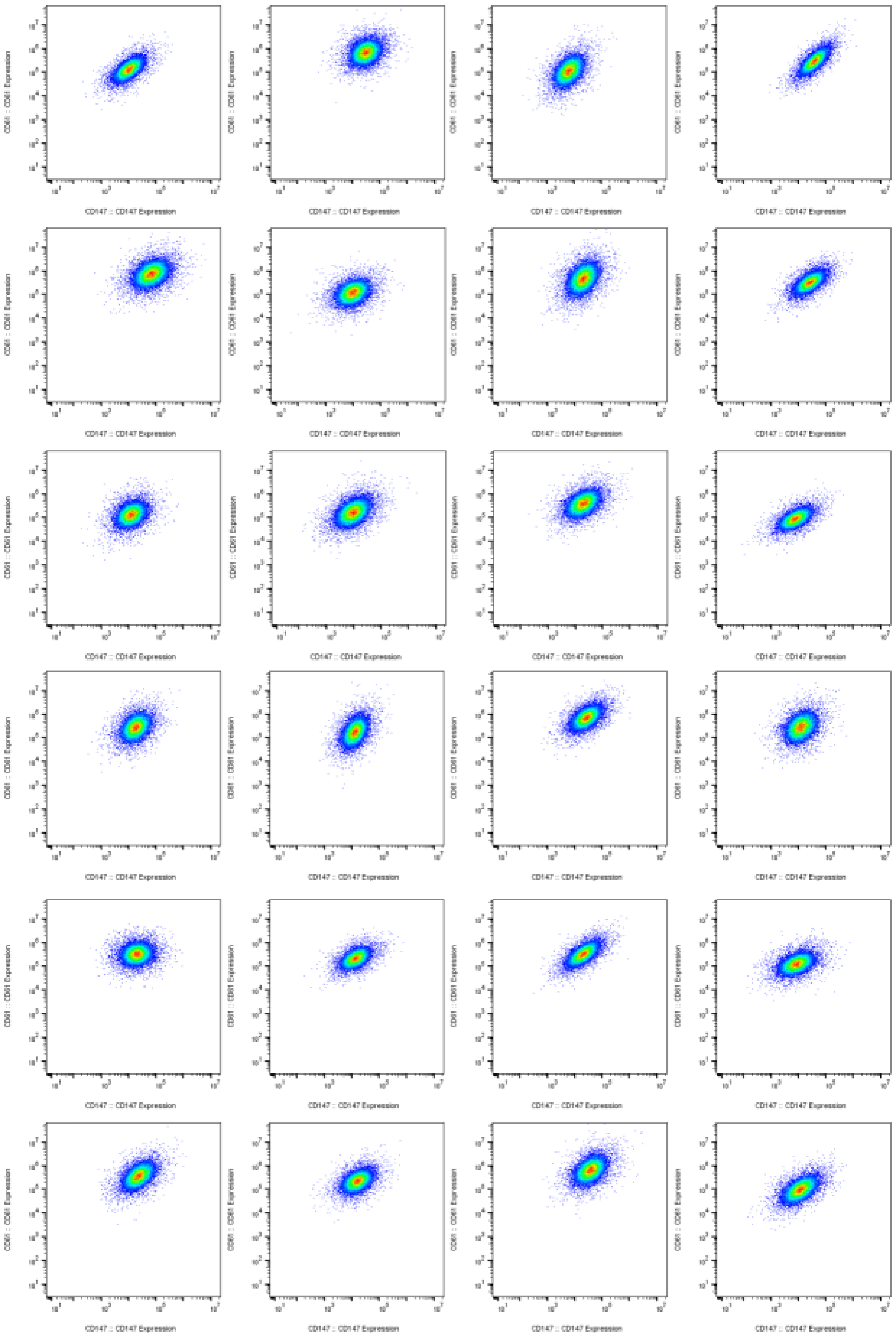
CD61 :: CD61 Expression
CD147 :: CD147 Expression

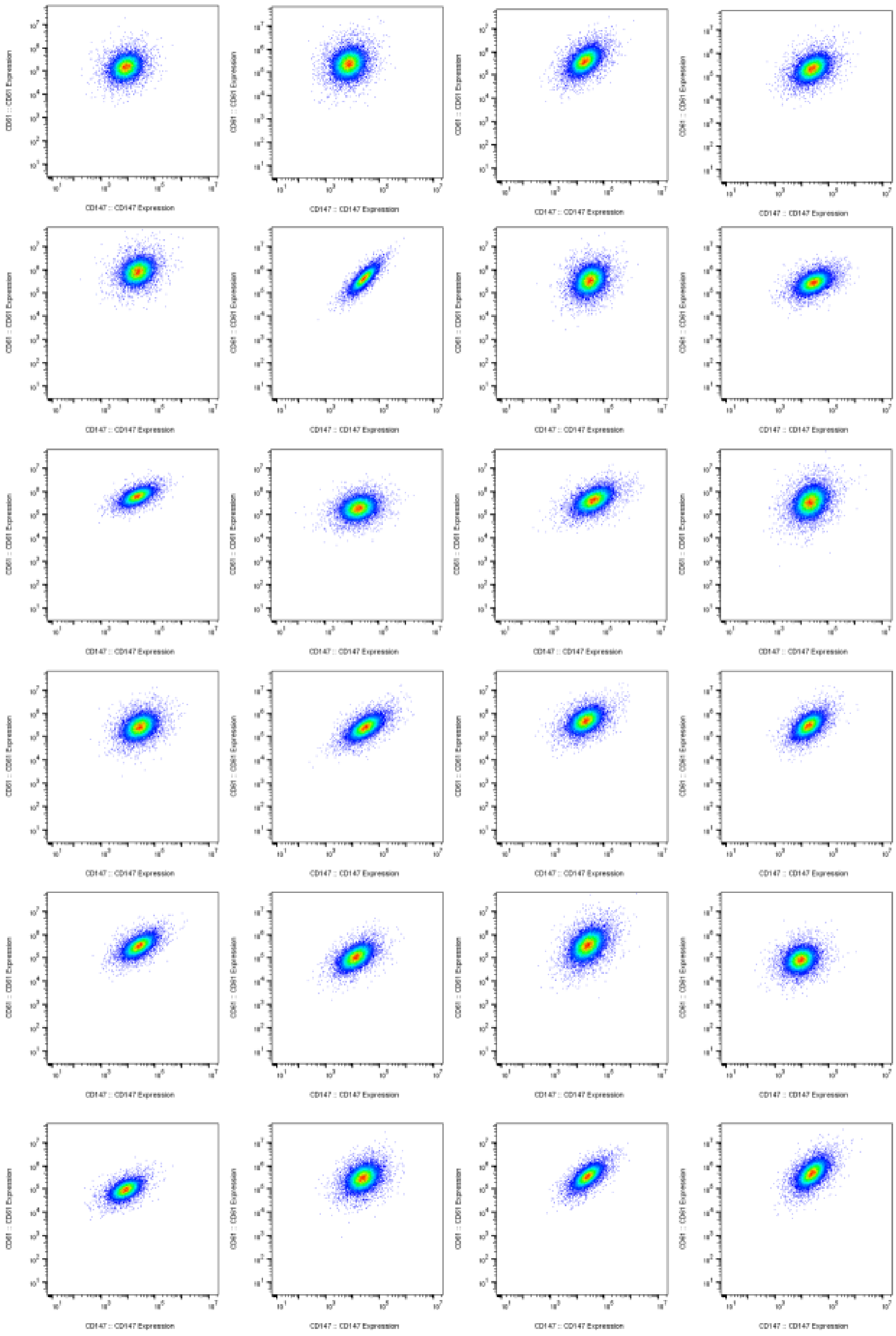
CD61 :: CD61 Expression
CD147 :: CD147 Expression

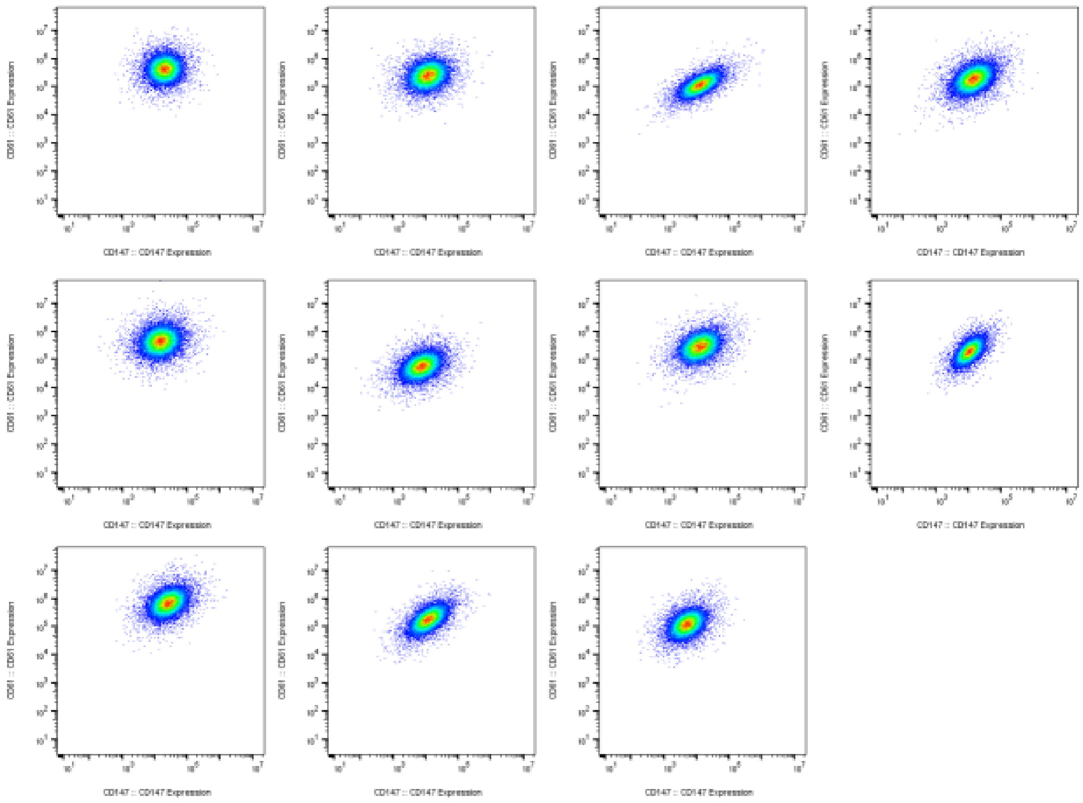

FigureS 1. Flow Cytometric Platelet Analysis of 90 Patients with Stable Angina Pectoris.

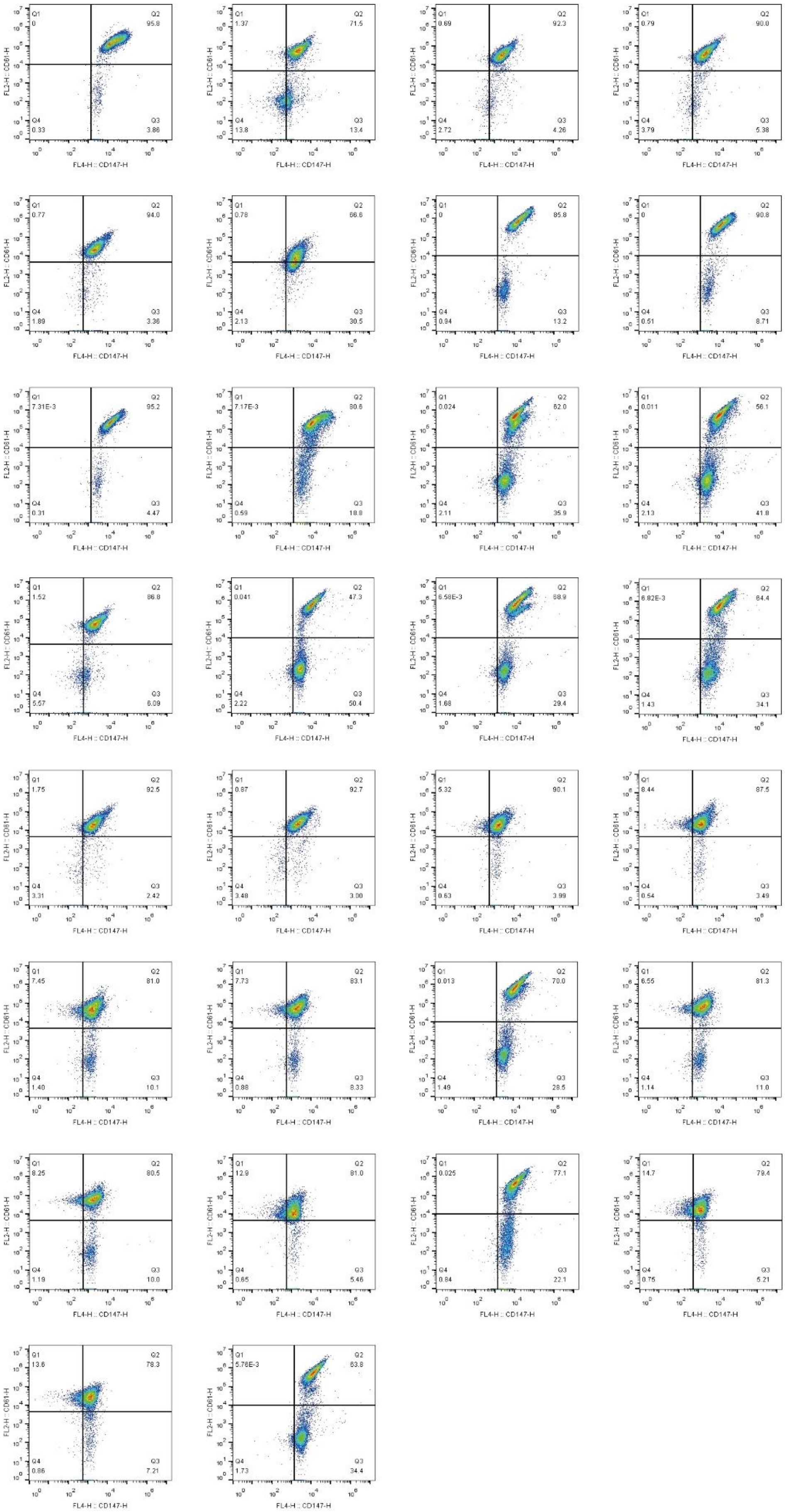
FL2-H :: CD61-H
FL4-H :: CD147-H
Q1 0 Q2 95.8 Q4 0.33 Q3 3.86
Q1 1.37 Q2 71.5 Q4 13.8 Q3 13.4
Q1 0.69 Q2 92.3 Q4 2.72 Q3 4.26
Q1 0.79 Q2 90.0 Q4 3.79 Q3 5.38
Q1 0.77 Q2 94.0 Q4 1.89 Q3 3.36
Q1 0.78 Q2 66.6 Q4 2.13 Q3 30.5
Q1 0 Q2 85.8 Q4 0.94 Q3 13.2
Q1 0 Q2 90.8 Q4 0.51 Q3 8.71
Q1 7.31E-3 Q2 95.2 Q4 0.31 Q3 4.47
Q1 7.17E-3 Q2 80.6 Q4 0.59 Q3 18.8
Q1 0.024 Q2 62.0 Q4 2.11 Q3 35.9
Q1 0.011 Q2 56.1 Q4 2.13 Q3 41.8
Q1 1.52 Q2 86.8 Q4 5.57 Q3 6.09
Q1 0.041 Q2 47.3 Q4 2.22 Q3 50.4
Q1 6.58E-3 Q2 68.9 Q4 1.68 Q3 29.4
Q1 6.82E-3 Q2 64.4 Q4 1.43 Q3 34.1
Q1 1.75 Q2 92.5 Q4 3.31 Q3 2.42
Q1 0.87 Q2 92.7 Q4 3.48 Q3 3.00
Q1 5.32 Q2 90.1 Q4 0.63 Q3 3.99
Q1 8.44 Q2 87.5 Q4 0.54 Q3 3.49
Q1 7.45 Q2 81.0 Q4 1.40 Q3 10.1
Q1 7.73 Q2 83.1 Q4 0.88 Q3 8.33
Q1 0.013 Q2 70.0 Q4 1.49 Q3 28.5
Q1 6.55 Q2 81.3 Q4 1.14 Q3 11.0
Q1 8.25 Q2 80.5 Q4 1.19 Q3 10.0
Q1 12.9 Q2 81.0 Q4 0.65 Q3 5.46
Q1 0.025 Q2 77.1 Q4 0.84 Q3 22.1
Q1 14.7 Q2 79.4 Q4 0.75 Q3 5.21
Q1 13.6 Q2 78.3 Q4 0.86 Q3 7.21
Q1 5.76E-3 Q2 63.8 Q4 1.73 Q3 34.4

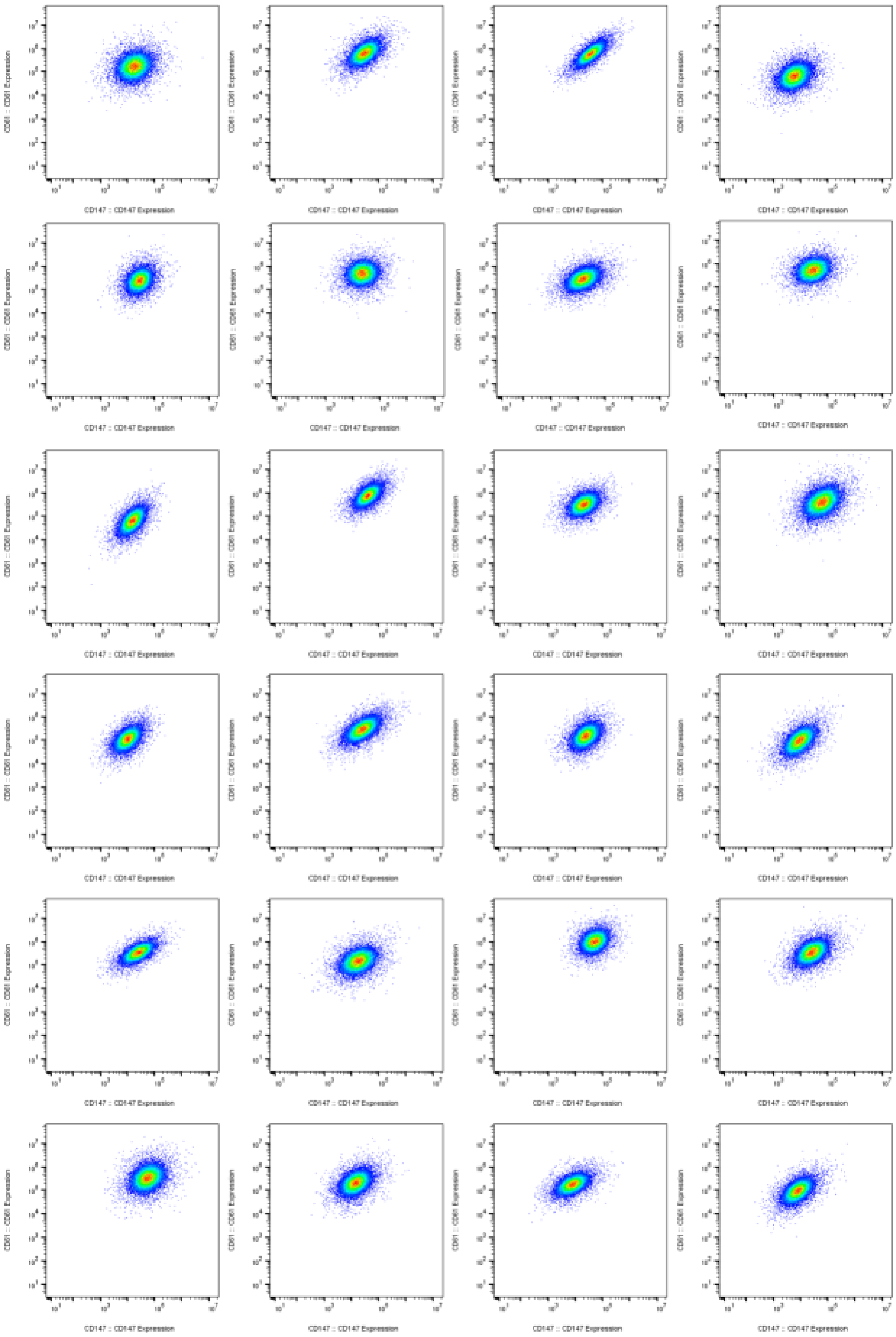
CD61 :: CD61 Expression
CD147 :: CD147 Expression

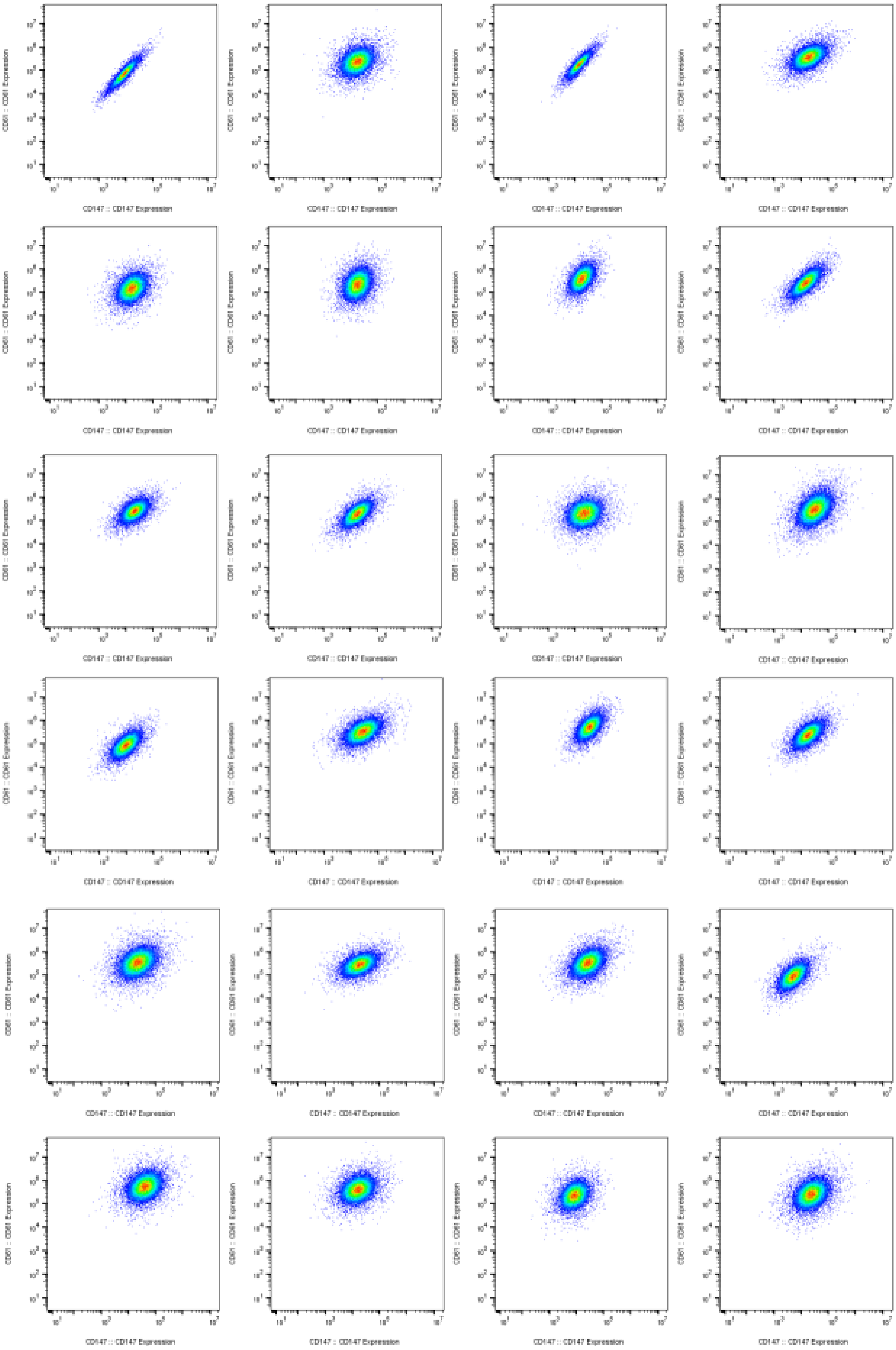
CD61 :: CD61 Expression
CD147 :: CD147 Expression

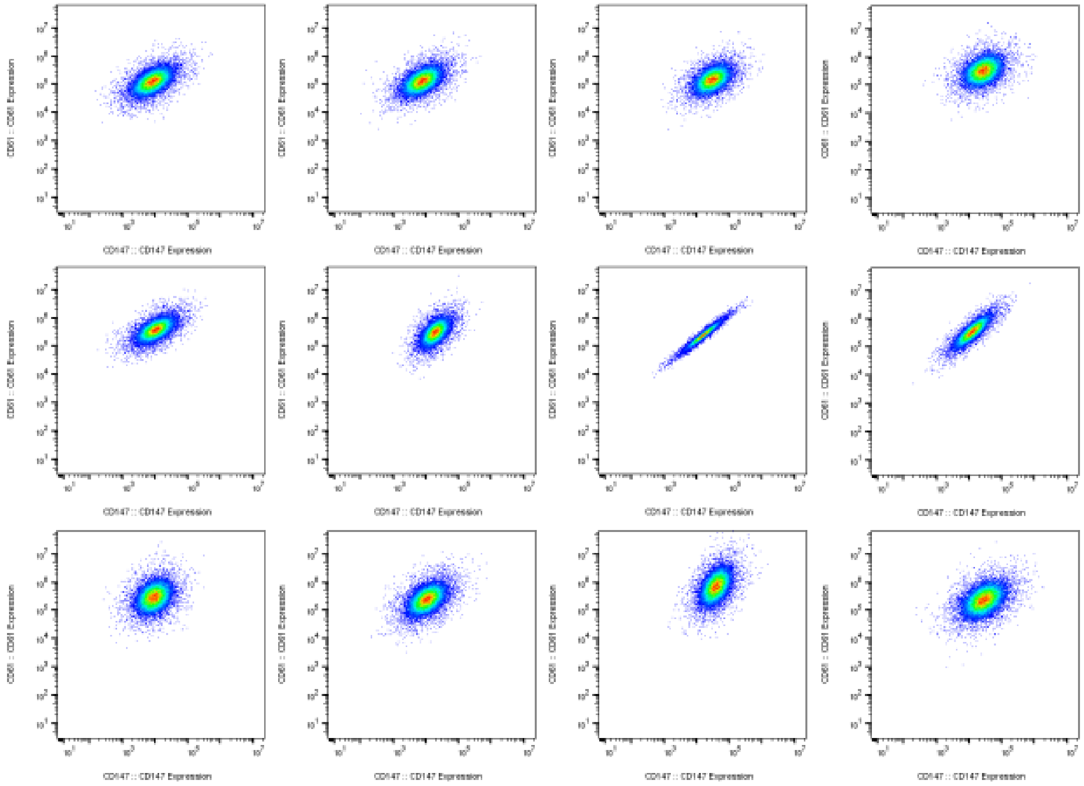

FigureS2. Flow Cytometric Platelet Analysis of 90 Patients with Acute Coronary Syndrome.
